# Effects of stigma on the quality of life in patients with epilepsy

**DOI:** 10.1186/s42494-024-00154-7

**Published:** 2024-04-03

**Authors:** Chunmei Hu, Yuping Zhao, Zheng Xiao

**Affiliations:** 1 Department of Neurology, The First Affiliated Hospital of Chongqing Medical University, NO.1 Youyi Rd., Chongqing, Yuzhong District 400016 China; 2 Department of Neurology, The People’s Hospital of Nanchuan, Chongqing, 408400 China

**Keywords:** Perceived stigma, Epilepsy, Quality of life

## Abstract

**Background:**

This study was aimed to evaluate the stigma and quality of life (QoL) in adult patients with epilepsy (PWEs) and explore the relationship between stigma and QoL.

**Methods:**

Two hundred and ninety-eight PWEs admitted to the Epilepsy Center of the First Affiliated Hospital of Chongqing Medical University during September 2020 and March 2021 were enrolled in this study. All participants completed self-reported questionnaires including the Stigma Scale for Epilepsy and the Quality of Life in Epilepsy Inventory-31 (QOLIE-31).

**Results:**

A total of 146 (49%) PWEs reported an experience of stigma. The total score of QOLIE-31 and the individual scores of seven subscales (worry about new seizures, emotion, well-being, energy and fatigue, cognitive impairment, medication effect, and social function) were significantly decreased in these patients (*P* < 0.001). Multivariate stepwise linear regression analysis showed that the annual household income per capita, the number of antiseizure medications and stigma had statistically significant effects on QoL (*P* < 0.05). Among them, stigma had the most significant negative effect.

**Conclusions:**

Nearly half of PWEs have experienced stigma. Stigma, lower household income per capita, and polypharmacy treatment are associated with poorer QoL. Stigma has the most obvious negative impact.

## Background

Epilepsy has become one of the major public health problems worldwide. The quality of life (QoL) among patients with epilepsy (PWEs) is affected by various factors [1, 2]. Early in 1995, a study in the United States found that the health-related QoL in PWEs was significantly lower than that in the healthy population [3]. Another cohort study in the UK further confirmed that, compared to the healthy population and patients with some other general chronic diseases such as diabetes, arthritis and migraine, PWEs have a poorer QoL [4, 5]. Multiple factors have been reported to affect the QoL of PWEs, such as seizure frequency, illness duration, number of antiseizure medications (ASMs), and adverse drug reactions [6–8]. Besides, psychosocial factors including stigma, anxiety, depression, economic status, and employment status, are reported to be more likely to affect the QoL of PWEs [7, 9].

The seizure attacks are usually unpredictable. The sudden loss of consciousness, limb convulsion, the subsequently altered psychological state, and occasional urinary incontinence, are causes of the feeling of stigma in PWEs [10, 11]. In addition, stigma has been associated with depression, anxiety, reduced self-esteem, reduced self-efficacy, and poor medication compliance, ultimately affecting the QoL [9, 12]. A huge body of research in European countries and the US has demonstrated that PWEs with feeling of stigma have a poorer QoL [13–15], and there is a negative correlation between stigma and QoL of PWEs [9, 13, 16]. In Asia, the word “epilepsy” is often perceived with a negative meaning. The situation of stigma is more prominent in PWEs. It is reported that in many Asian countries, the presence of stigma also remarkably affects the QoL of PWEs [17]. Studies during the 1980s in China estimated that up to 89% of PWEs had experienced stigma [18]. A recent study found that even when patients are seizure-free with complete discontinuation of ASMs, stigma may still persist and lead to a lower QoL [19].

In China, studies on stigma usually focused on the public attitudes toward PWEs [20, 21]. Few studies have reported the impact of stigma on the QoL of PWEs. In this study, we set out to investigate the current situation of stigma toward and QoL in adult PWEs in southwest China and explore the relationship between stigma and QoL**.**

## Methods

### Participants

PWEs who regularly visited the epilepsy clinic of the First Affiliated Hospital of Chongqing Medical University were recruited between September 2020 and March 2021. The study protocol was approved by the Ethics Committee of the First Affiliated Hospital of Chongqing Medical University. PWEs were included if they: (1) were diagnosed with epilepsy according to the International League Against Epilepsy (ILAE) criteria [22], (2) were aged ≥ 18 years, (3) were on stable doses of one or more ASMs over 30 days, (4) had received at least 6 years of education and were able to complete the questionnaires independently, and (5) signed an informed consent form.

PWEs were excluded if they: (1) were unable to understand the questionnaire, or (2) had obvious neurological/psychiatric disorders (aphasia, schizophrenia, etc.), which may lower the accuracy of the survey results.

### The Stigma Scale for Epilepsy (SSE)

The SSE is a three-item self-rating instrument developed by Jacoby et al. in 1994 according to a stigma scale originally used in stroke patients [23]. The SSE has been confirmed as a reliable measurement in PWEs, with reported α coefficients of 0.8222 [24] and 0.7723 [25]. According to SSE, participants were asked whether (1) they felt that someone else was uncomfortable with them, (2) they were treated as inferior by others, and (3) they were excluded by others because of epilepsy. Each of the three items requires a simple “yes” or “no” response. Patients were scored 0 for no “yes”, 1 for one “yes”, 2 for two “yes” and 3 for all “yes”. Accordingly, the PWEs were categorized as having no (total score 0), mild (total score 1), moderate (total score 2) or severe (total score 3) feeling of stigma. A higher score indicates more severe stigma.

### The quality of life in epilepsy inventory-31 (QOLIE-31)

The QOLIE-31 instrument was used to evaluate the QoL of PWEs. This scale covers 31 items in total and consists of 7 subscales including worry about new seizures, emotion, well-being, energy and fatigue, cognitive impairment, medication effect, and social function [26]. Seven individual scores (per subscale) and a total (composite) score are yielded. A higher score indicates a better QoL.

### Statistical analysis

Statistical analysis was performed using SPSS version 24.0. Quantitative variables are presented as mean ± SD and qualitative variables as frequencies and percentages. Cronbach's α coefficient was used to analyze the reliability of the scale. The Kolmogorov–Smirnov (K-S) test was used to determine if a variable was normally distributed. In the univariate analysis, *t*-test or one-way ANOVA was used to compare the means. The Chi-square test and Fisher's exact test were used to compare percentages between groups. Spearman correlation was used to analyze the correlations between continuous variables, with significance level set as *P* < 0.05 (two-tailed). To explore the risk factors for QoL in PWEs, variables with *P* ≤ 0.1 in the univariate analysis were selected as the independent variables. Then the QoL was used as the dependent variable, and multiple linear regression (stepwise regression) was performed in multivariate analysis by using SPSS version 24.0. The statistical significance of the results was determined based on 95% confidence intervals. *P* < 0.05 (two-tailed) was considered as significantly different.

## Results

A total of 406 adult PWEs were screened, and 95 patients who could not fully understand the content of the questionnaire as well as 13 patients with obvious neurological disorders were excluded. Finally, 298 patients were included.

We analyzed the clinical data by using stigma as the independent variable. The Cronbach's α coefficient for the SSE in this study was 0.71, signifying a commendable level of scale reliability.

### Distribution of stigma scores and differences in demographic and clinical characteristics

We analyzed the clinical data by using stigma as the independent variable. The demographic and clinical characteristics of the 298 PWEs are shown in Table 1.

**Table 1:** Demographic and clinical characteristics of the people with epilepsy

	Total sample (*n* = 298)
Age (years), mean ± SD	36.95 ± 16.38
Sex,* n* (%)	
Male	158 (53.0%)
Female	140 (47.0%)
Domicile,* n* (%)	
Urban area	133 (44.6%)
Rural area	165 (55.4%)
Marital status,* n* (%)	
Single	121 (41.6%)
Married	177 (59.4%)
Education levels,* n* (%)	
Primary school	36 (12.1%)
Junior high school	109 (36.6%)
High school	92 (30.9%)
University and above	61 (20.5%)
Employment status,* n* (%)	
Students	34 (11.4%)
Unemployed	116 (38.9%)
Part-time employee	21 (7.0%)
Full-time employee	106 (35.6%)
Retired	21 (7.0%)
Income level (yuan),* n* (%)	
< 10,000	154 (51.7%)
10,000–50,000	110 (36.9%)
> 50,000	34 (11.4%)
Age at onset (years), mean ± SD	28.49 ± 19.18
Duration of epilepsy (years), mean ± SD/ median	8.63 ± 8.89
Seizure frequency (within 6 months), mean ± SD	17.36 ± 92.43
Epilepsy types [27],* n* (%)	
Focal epilepsies	48 (16.1%)
Generalized epilepsies	248 (83.2%)
Unknown epilepsies	2 (0.7%)
ASMs therapy regimen,* n* (%)	
Monotherapy	229(76.8%)
Polytherapy	69 (23.2%)
History of febrile convulsions,* n* (%)	
Yes	22 (7.4%)
No	276 (92.6%)
Causes of epilepsy	
Known	63 (21.1%)
Unexplained	235 (78.9%)

*Note*: “Income level” refers to the average annual income per person

**Fig. 1:**
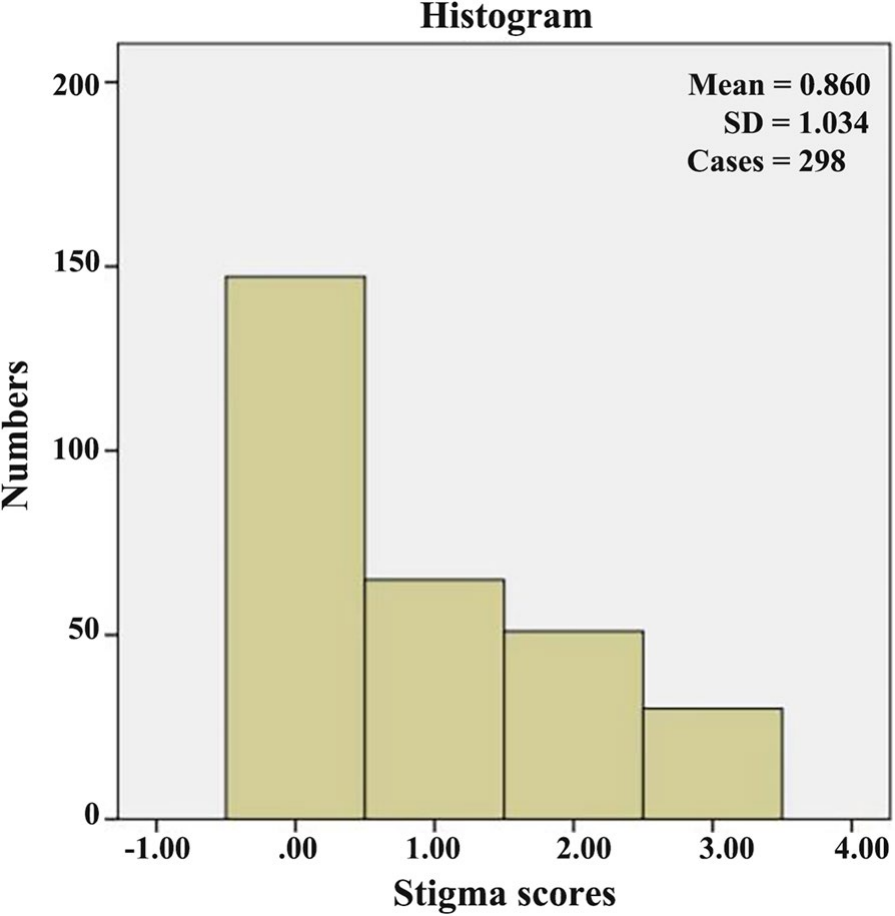
The distribution of Stigma Scale for Epilepsy scores among the 298 patients with epilepsy

Specifically, 146 out of the 298 (49.0%) PWEs reported to have experienced stigma (SSE total score ≥ 1), and 30 (10.1%) of them experienced the highest stigma (SSE total score 3) (Fig. 1). The mean score of SSE was 0.86 ± 1.034. The patients were categorized as stigmatized (SSE total score ≥ 1) and non-stigmatized (SSE total score 0). As shown in Table 2, the seizure frequency in the stigmatized patients was significantly higher than that in the non-stigmatized patients (*P* = 0.003). In addition, the history of febrile convulsions was also significantly different between the two groups (*P* = 0.006). There were no significant differences in other parameters such as age, sex, marital status, residence, education level, employment status, income level, age at onset, duration of epilepsy, and seizure frequency between the two groups.

**Table 2:** Comparison of demographic and clinical characteristics between PWEs with and without experience of stigmatization

	Stigmatized (*n* = 146)	Non-stigmatized (*n* = 152)	*P* value
Age (years), mean ± SD/ median	38.23 ± 16.88/33.00	35.72 ± 15.85/32.00	0.193^a^
Sex,* n* (%)			0.286^b^
Male	82 (56.2%)	76 (50.0%)	
Female	64 (43.8%)	76 (50.0%)	
Domicile,* n* (%)			0.668^b^
Urban area	67 (45.9%)	66 (43.4%)	
Rural area	79 (54.1%)	86 (56.6%)	
Marital status,* n* (%)			0.213^b^
Single	54 (37%)	67 (44.1%)	
Married	92 (63.0%)	85 (55.9%)	
Education level,* n* (%)			0.082^b^
Primary school	20 (13.7%)	16 (10.5%)	
Junior high school	62 (42.5%)	47 (30.9%)	
High school	37 (25.3%)	55 (36.2%)	
University and above	27 (18.5%)	34 (22.4%)	
Employment status,* n* (%)			0.809^b^
Students	14 (9.6%)	20 (13.2%)	
Unemployed	59 (40.4%)	57 (37.5%)	
Part-time employee	12 (8.2%)	9 (5.9%)	
Full-time employee	51 (34.9%)	55 (36.2%)	
Retired	10 (6.8%)	11 (7.2%)	
Income level (yuan),* n* (%)			0.814^b^
< 10,000	73 (50.0%)	81 (53.3%)	
10,000–50,000	55 (37.7%)	55 (36.2%)	
> 50,000	18 (12.3%)	16 (10.5%)	
Age at onset (years), mean ± SD/ median	29.02 ± 20.63/23.50	27.97 ± 17.73/23.00	0.851^a^
Duration of epilepsy (years), mean ± SD/ median	9.34 ± 9.08/7.00	7.94 ± 8.68/4.00	0.151^a^
Seizure frequency (within 6 months), mean ± SD/ median	25.73 ± 126.05/2.00	9.32 ± 37.62/1.00	**0.003**^**a**^
Epilepsy types,* n* (%)			0.320^b^
Focal epilepsies	22 (15.1%)	26 (17.1%)	
Generalized epilepsies	122 (83.6%)	126 (82.9%)	
Unknown epilepsies	2 (1.4%)	0 (0.0%)	
ASM therapy regimen,* n* (%)			0.154^b^
Monotherapy	107 (73.3%)	122 (80.3%)	
Polytherapy	39 (26.7%)	30 (19.7%)	
History of febrile convulsions,* n* (%)			**0.006**^**b**^
Yes	17 (11.6%)	05 (3.3%)	
No	129 (88.4%)	147 (96.7%)	
Causes of epilepsy			0.145^b^
Known	36 (24.7%)	27 (17.8%)	
Unexplained	110 (75.3%)	125 (82.2%)	

Note: “Income level” refers to the average annual income per person. Data in bold represent statistically significant difference (*P* < 0.05)

^a^ Mann–Whitney U test. ^b^ Chi-square test

### QoL scores in stigmatized and non-stigmatized PWEs

We then analyzed the differences in QoL between stigmatized and non-stigmatized PWEs with Student’s *t*-test. Compared to the non-stigmatized PWEs, the stigmatized PWEs had a significantly lower total QoL score (*P* < 0.001) (Table 3). The individual scores of 7 subscales were also significantly lower in the stigmatized PWEs (*P* < 0.001).

**Table 3:** Differences in QoL between patients with and without stigma

	Pooled score	PWEs with experience of stigmatization				PWEs without experience of stigmatization				Statistical analysis	
		M1	SD	M2	M3	M1	SD	M2	M3	z	*P*
Overall quality of life	63.95 ± 15.34	57.09	13.92	56.72	110.44	70.54	13.70	70.41	187.02	-7.67	** < 0.001**
Social function	68.52 ± 23.66	60.77	20.87	62.00	117.54	75.96	23.85	85.00	180.19	-6.28	** < 0.001**
Energy	62.53 ± 20.63	55.48	19.38	55.00	119.02	69.31	19.54	70.00	178.78	-6.01	** < 0.001**
Medication effects	57.31 ± 23.54	51.11	22.24	52.77	126.59	63.27	23.29	61.10	171.51	-4.52	** < 0.001**
Cognitive	64.14 ± 22.87	58.20	21.30	58.61	127.26	69.85	22.94	68.75	170.86	-4.37	** < 0.001**
Emotional	65.87 ± 20.27	58.37	19.05	60.00	116.30	73.08	18.79	72.00	181.38	-6.53	** < 0.001**
Seizure worry	48.66 ± 24.81	39.39	19.20	43.66	114.92	57.56	26.34	58.68	182.71	-6.79	** < 0.001**
Well-being	64.85 ± 16.26	60.67	16.23	62.50	128.98	68.87	15.29	72.50	169.21	-4.06	** < 0.001**

*Note*: *M1* means, *M2* median, *M3* average rank, *SD* standard deviation

### Univariate analysis of the correlation between demographic/clinical characteristics and QoL in PWEs

We then explored the factors affecting the QoL of PWEs by using QoL as the dependent variable. First, we analyzed the differences in the demographics (sex, place of residence, marital status, etiology of epilepsy, ASM therapy regimen, and history of febrile convulsions) of PWEs by using Student’s *t*-test. The results showed that sex, place of residence, marital status and etiology of epilepsy were not correlated with the QoL of PWEs. However, the history of febrile seizures (*P* = 0.021) and the number of ASMs (*P* < 0.001) were correlated with QoL (Table 4). The results suggest that PWEs with no history of febrile seizures or receiving monotherapies might have a better QoL.

**Table 4:** Factors associated with the quality of life of PWEs

Univariate analysis								
Variables					Post-hoc comparisons			
	*n*	M ± SD	T	*P*	*P*			
Sex								
Male	158	63.95 ± 14.98	-0.01	0.992				
Female	140	63.96 ± 15.80						
Domicile								
Rural	165	62.94 ± 15.94	1.27	0.205				
City	133	65.21 ± 14.53						
Marital status								
Single	121	63.61 ± 15.53	-0.32	0.746				
Married	177	64.19 ± 15.25						
Causes of epilepsy								
Known	63	62.65 ± 16.51						
Unknown	235	64.30 ± 15.03	0.757	0.449				
ASM therapy regimen								
Monotherapy	229	65.96 ± 14.79	4.22	** < 0.001**				
Polytherapy	69	57.31 ± 15.36						
History of febrile convulsions								
Yes	22	56.68 ± 14.74	-2.33	**0.021**				
No	276	64.53 ± 15.26						
Education level			1.09	0.356				
Primary school	36	65.42 ± 13.08						
Junior high school	109	61.85 ± 14.79						
High school	92	65.23 ± 16.73						
University and above	61	64.92 ± 15.32						
Epilepsy types			0.66	0.518				
Focal epilepsies	48	65.51 ± 16.00						
Generalized epilepsies	248	63.73 ± 15.18						
Unknown epilepsies	2	54.41 ± 25.07						
Employment status			3.99	**0.004**				
Students	34	70.93 ± 12.54						
Unemployed	116	60.20 ± 15.57			**0.003**			
Part-time employee	21	64.07 ± 16.09			0.471	0.814		
Full-time employee	106	65.66 ± 15.09			0.388	0.056	0.992	
Retired	21	64.69 ± 14.41			0.567	0.717	1.000	0.999
Income level (yuan)			5.16	**0.006**				
< 10,000	154	61.25 ± 14.51						
10,000–50,000	110	66.59 ± 16.47			**0.014**			
> 50,000	34	67.69 ± 15.34			0.066	0.927		
	Spearman correlation			*P*				
Age (years)	0.012			0.836				
Age at onset (years)	0.034			0.554				
Duration of Epilepsy (years)	-0.031			0.590				
Seizure frequency	-0.337			** < 0.001**				

*Note*: “Income level” refers to the average annual income per person. Data in bold represent statistically significant difference (*P* < 0.05)

With one-way ANOVA, we analyzed the differences in QoL between different PWE groups in terms of demographic parameters such as education level, epilepsy type, employment status, or income level. The results showed no significant differences in the QoL among PWEs with different education levels or different epilepsy types. However, there were significant differences in the QoL among PWEs with different employment status (*P* = 0.004) or income levels (*P* = 0.006) (Table 4). Our results implied that PWEs who are unemployed and have an annual income of less than 10,000 yuan may experience a worse QoL.

We further used Spearman correlation to analyze the correlation between the QoL of PWEs and age, age at onset, duration of disease, and seizure frequency. A negative correlation was found between QoL and seizure frequency (*P* < 0.001), indicating that the higher the seizure frequency, the worse the QoL of PWEs.

### Multiple linear regression analysis of the correlation between stigma/demographic/clinical characteristics and QoL of PWEs

We further analyzed the correlated variables for the QoL of PWEs with stepwise multiple linear regression. Variables with *P* ≤ 0.1 in univariate analyses were included in the multivariate analyses. The results showed that there was no collinearity among the independent variables. The adjusted *R*^2^ was 0.30 and *F *(df1, df2) was 43.42, indicating that the regression model fit the data well (*P* < 0.001). Three variables including annual household income per capita, number of ASMs, and stigma, had a significant impact on QoL (*P* < 0.05) (Table 5). The PWEs with a lower annual household income per capita, a combinational drug therapy, and stigma would have a lower QoL. The corresponding regression equation was as follows: QoL = 0.17 × (annual household income per capita) − 0.17 × (number of ASMs) − 0.48 × (SSE total score). According to the non-standardized coefficient, it was calculated that stigma has the greatest negative impact on QoL.

**Table 5:** Regression analysis for the quality of life in PWEs

Multivariate analysis						
Variables	Non-standardized coefficient		Standardized coefficient			
	B	SE	β	t	*P*	VIF
Intercept	71.45	3.03		23.61	< 0.001	
Income level	3.86	1.09	0.17	3.54	< 0.001	1.01
ASM therapy regimen	-46.10	1.78	-0.17	-3.42	0.001	1.02
SSE	-7.13	0.73	-0.48	-9.82	0.001	1.01

*F* = 43.42; *P* < 0.001; D-W = 1.795; *R*^2^ = 0.30

*Note*: “Income level” refers to the average annual income per person. *B* regression coefficients; *SE* standard error; *VIF* variance inflation factor

## Discussion

Improving the QoL of PWEs is one of the main targets in the treatment of epilepsy [28]. The present study investigated the status of stigma and QoL of adult PWEs in southwest China. We found that nearly 49.0% of PWEs reported stigma. Compared to the non-stigmatized PWEs, the stigmatized PWEs had a significantly lower total score of QOLIE-31 and individual scores of the seven subscales. Further analysis showed that stigma, annual household income per capita, and the number of ASMs had significant effects on the QoL of PWEs, and stigma had the most significant negative effect. To our knowledge, this is the first study on the quantitative relationship between stigma and QoL of PWEs in China.

Epilepsy-related stigma is commonly seen in PWEs worldwide. In 2018, WHO released a report highlighting the influence of stigma on the physical, mental, and social health of PWEs [29]. One European survey showed that more than 50% PWEs had experienced stigma [30]. Studies in Asian countries have reported stigma experienced by 9% to 89% patients [17]. A study in the 1980s on epilepsy stigma in China found that approximately 89% of PWEs and 76% of their family members experienced stigma [31]. In the present study, 146 out of 298 (49.0%) adult PWEs reported experience of stigma. Apparently, this percent was much lower than that reported in other published articles. The discrepancy could be due to the different methodologies in our study versus in previous studies, and study populations from different regions. Another possible reason for this discrepancy might be the improved online and offline education on epilepsy among PWEs, their family members, and the public in recent years, with increased availability of psychological counseling services in this field [31].

It has been reported that epilepsy is the second leading cause of stigma following AIDS among various chronic diseases [32]. A large number of studies have revealed that stigma has a significant impact on the QoL of PWEs [9, 33, 34]. Here we found that compared to the non-stigmatized PWEs, the stigmatized PWEs had an overall lower QoL concerning worry about new seizures, emotion, well-being, energy and fatigue, cognitive impairment, medication effect, and social function. This was consistent with the results reported in previous literature [35, 36]. We further developed a regression equation, which implied that PWEs with a lower household income per capita, a combinational drug therapy, and stigma would have worse QoL. Notably, among the three variables, stigma had the greatest negative impact on QoL. Previous studies have revealed that stigma is associated with many social-psychosocial factors [37]. And social-psychological factors also have an important impact on the QoL of PWEs [9, 38], even in PWEs with well-controlled seizures [28]. Thess reports, together with our findings, suggest that stigma has a negative impact on the QoL of PWEs. Some measures have been reported to alleviate the stigma, such as strengthening psychological counseling and public education to improve the awareness of epilepsy [31, 39].

In this study, we also found that PWEs with a high frequency of seizures were more likely to experience stigma. Although the variables related to stigma in PWEs are controversial in different studies, the seizure frequency is considered the most consistent predictor of stigma among studies [40, 41]. Medical services aiming to improve the diagnosis and treatment of epilepsy may be another effective way to overcome the epilepsy-associated stigma [31]. Therefore, standard diagnosis and treatment of epilepsy to control epileptic seizures may be another strategy to mitigate stigma and improve the QoL of PWEs.

In addition, our findings reported that the number of ASMs and the annual household income per capita could also influence the QoL of PWEs. The PWEs with polymedication treatment and low annual household income per capita tend to have lower QoL. Concerning the effect of number of ASMs on QoL, previous studies have reported inconsistent results. Alexander et al. revealed the correlation between QoL and the number of ASMs [42]. However, Millul et al. did not find this correlation between QoL and the number of ASMs [43]. We speculated that the polymedication treatment might result in increased side effects and more medical expenses in our study population, thus leading to lower QoL. As to the financial issue, many studies have revealed that PWEs with higher incomes would have a higher QoL than those with lower incomes. In China, it is estimated that the annual medical expenses and the loss of productivity accounted for more than half of their average annual income, posing an immense economic burden on PWEs [44]. Comparatively, PWEs with high incomes seem to have less of these difficulties and acquire a higher QoL. Therefore, appropriate health policies are needed to reduce the financial burden and improve the QoL of PWEs.

Although our current study focused solely on the perceived stigma of PWEs, enacted stigma, which often refers to episodes of discrimination and misconduct, also has a wide and significant impact on the QoL of PWEs. Recently, more and more researchers are working to prevent and eliminate stigma from the perspective of the public [45]. Efforts to de-stigmatize epilepsy in the society such as public education and formulating legal frameworks, have been proven valuable strategies to reduce epilepsy stigma and promote the QoL of PWEs [45]. Taken together, de-stigmatization is a complicated process involving comprehensive strategies targeting both PWEs and the public.

The present study still had some limitations. First, the three-item SSE scale is a classic, relatively simple survey questionnaire which was originally developed for English speakers. Although it has been used in studies on PWEs in China [46], its applicability in Chinese PWEs still needs to be further verified. Second, for some reasons such as unclear description of the symptoms at onset of epilepsy, lack of expertise, and lack of EEG recordings, the patients with generalized epilepsy were far more than those with focal epilepsy in this study, which was in contrast with some clinical practices. Therefore, there might be a selection bias which may influence the results of the study. Third, in the present study, we aimed to investigate the relationship between stigma and QoL. Some psychological factors including depression and anxiety were not included. In future studies, psychological factors should be included to analyse their effects on the QoL in PWEs. Finally, this was a cross-sectional study with participants from a single center, and the sample size was limited. Multicenter longitudinal studies with large sample sizes are necessary in the future to further confirm our findings.

## Conclusions

In conclusion, our study found that the felt stigma, the number of ASMs, and the annual household income per capita were significantly related to the QoL of PWEs. Among them, the felt stigma had the greatest negative impact on QoL. We propose that positive preventive strategies for stigma could be an important way to improve the QoL of PWEs.

## Data Availability

The data supporting the findings of this article are provided within the manuscript and are available from the corresponding author on reasonable request.

## Abbreviations

PWE: Patient with epilepsy
SSE: The Stigma Scale for Epilepsy
QOLIE-31: The quality of life in epilepsy inventory-31
QoL: Quality of life
